# A fusion ORF3a-E subgenomic RNA involved in SARS-CoV-2 infection efficacy by influencing cellular protein synthesis

**DOI:** 10.3389/fimmu.2025.1619538

**Published:** 2025-08-15

**Authors:** Yifan Zhang, Jing Li, Xinglong Zhang, Xin Zhang, Jiali Li, Heng Li, Xin Zhao, Zihan Zhang, Yingyan Li, Keqi Chen, Shasha Peng, Haijing Shi, Longding Liu

**Affiliations:** ^1^ Institute of Medical Biology, Chinese Academy of Medical Sciences and Peking Union Medical College, Kunming, Yunnan, China; ^2^ Key Laboratory of Systemic Innovative Research on Virus Vaccine, Chinese Academy of Medical Sciences, Kunming, Yunnan, China; ^3^ Yunnan Key Laboratory of Vaccine Research and Development for Severe Infectious Diseases, Kunming, Yunnan, China

**Keywords:** SARS-CoV-2, subgenomic RNA, ribosome, viral replication, single-cell sequence

## Abstract

Subgenomic RNAs (sgRNAs) are discontinuous transcription products of severe acute respiratory syndrome coronavirus 2 (SARS-CoV-2) that are involved in viral gene expression and replication, but their exact functions are still being studied. Here, we report the identification of a nested ORF3a-sgRNA, the fusion ORF3a-E-sgRNA, which is involved in the infection process of SARS-CoV-2. This sgRNA encodes both ORF3a and E and can be detected throughout the viral life cycle in SARS-CoV-2-infected cells with high copy numbers. ORF3a-E-sgmRNA guides ORF3a translation and promotes the expression of cellular ribosomal protein S3 (RPS3), increasing translation levels. Single-cell sequencing of a SARS-CoV-2-infected human bronchial epithelial cell line (16HBE) revealed that maintenance of this stable translational environment by ORF3a-E-sgmRNA is important for SARS-CoV-2 assembly and release capabilities and is also beneficial for viral evasion of host innate immunity. More importantly, the transcription level of ORF3a-E-sgRNA may contribute to differences in infection processes between the Wuhan strain and the XBB strain of SARS-CoV-2.

## Graphical Abstract

Image created with BioRender (https://biorender.com/).

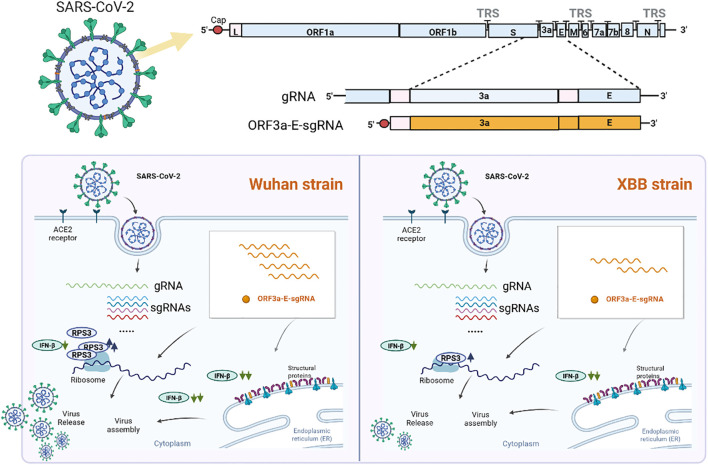

## Introduction

Subgenomic RNA (sgRNA) is produced by discontinuous transcription from genomic RNA via the replication-transcription complex (RTC) and the transcription regulatory sequence (TRS). The SARS-CoV-2 genome contains 14 open reading frames (ORFs) that are preceded by the TRS body sequence (TRS-B). Additionally, the TRS leader sequence (TRS-L) is located after the conserved 5′ leader sequence, with a core ACGAAC sequence that can be used for sgRNA detection. When the RTC encounters TRS-B, it performs discontinuous transcription with TRS-L, generating different sgRNAs of different sizes (1–3). In accordance with the transcription strategy of CoV, sgRNAs adopt a nested transcription mode, including several ORFs in the transcription sequence. For example, each sgRNA should contain at least the N-ORF during the transcription process (4, 5). To date, nine types of sgRNAs have been identified, including S, ORF3a, E, M, ORF6, ORF7, ORF8, N and ORF10 (expression unverified) during SARS-CoV-2 replication (6, 7), but the existence of fused forms of sgRNAs and the role of these fusion sgRNAs in virus replication and evolution during SARS-CoV-2 infection are unclear. sgRNAs and their encoded proteins are involved throughout the viral lifecycle, from infection to release. This is closely related to the ability of SARS-CoV-2 to rapidly replicate during infection, as these sgRNAs provide guidance for virus synthesis and helping the virus assemble into complete viral particles. The proximity of TRS-L and TRS-B sequences during discontinuous transcription increases the recombination frequency, increasing the synthesis speed of sgRNAs and aiding virus functions (6, 8). Four sgRNAs encode the spike protein (S), nucleocapsid protein (N), membrane protein (M), and envelope protein (E); the proteins translated from these sgRNAs encapsulate genomic RNA (gRNA) to form new viral particles that are released by cells. The remaining sgRNAs encode viral accessory proteins, which are a highly variable set of virus-specific proteins that help regulate the host response to infection. The sgRNAs of SARS-CoV-2 can assist in viral evasion of host immune defenses during infection, such as those encoding N, ORF9b, and ORF6, which influence the transcription of IFN-stimulated genes (ISGs) and accelerate viral replication via increases in the corresponding RNA and protein levels (9–11). Upon infection, SARS-CoV-2 may impact the host internal translation environment, usually by inhibiting the host’s translation activity, and the sgRNAs continue to initiate translation because of the presence of a leader sequence in the 5’ UTR.

SARS-CoV-2 variants have led to multiple waves of infection worldwide, such as the Alpha (B.1.1.7) variant from the UK in September 2020, the Beta (B.1.351) variant from South Africa in October 2020, the Gamma (P.1) variant from Brazil in November 2020, the Delta (B.1.617.2) variant from India in April 2021, and the Omicron (B.1.1.529) variant, which has been circulating since November 2021. Research indicates that as these variants evolve, they undergo significant changes in replication capacity, pathogenicity, and transmissibility (12). For example, in transgenic mouse and hamster models expressing human ACE2 receptors, the Alpha and Delta variants exhibited extensive replication across multiple organs. Moreover, the Omicron variant, although it spread more rapidly than did the Delta variant, shows substantially reduced infectivity and pathogenicity (13–15). Several factors contribute to the varying transmission and replication capabilities among these variants. These variants can enhance their ability to enter cells by improving fusion with the host cell membrane, better interacting with ACE2 receptors of other species, enabling cross-species transmission, and partially evading preexisting immunity within populations (16). Furthermore, variations in sgRNA frequency, the proteins they encode, and host cell activity levels can limit the resources available for viral particle formation, thus reducing replication capabilities (17). Studies suggest that infections with the Alpha variant have increased sgRNA abundance, which is closely tied to host cellular activities (18, 19). Therefore, the roles and functions of sgRNAs are critical during SARS-CoV-2 replication and significantly influence viral replication efficiency.

In our study, we investigated the discontinuous transcription of SARS-CoV-2 sgRNAs and discovered that sgRNAs indeed undergo discontinuous transcription, but only ORF3a-E-sgRNA exists as a nested fusion RNA among sgRNAs detected during the SARS-CoV-2 transcription process. Our results indicate that ORF3a-E-sgRNA notably recruits more of the cellular ribosomal protein S3 (RPS3) and binds with eukaryotic translation initiation factor 4E (eIF4E) compared to ORF3a-sgRNA. Notably, we observed significant differences in virus replication and host translation levels between 16HBE cells infected with the original 2019 Wuhan strain and those infected with the 2022 Omicron variant. Among them, ORF3a-E-sgRNA impacts the protein synthesis pathway during infection. Additionally, ORF3a-E-sgRNA seems to impair the host antiviral immune response to some extent, fostering a more favorable environment for SARS-CoV-2 replication.

## Methods

Animal study: Healthy male Chinese macaques aged 8–12 months were randomly assigned to either a normal control group or a virus challenge group. The virus challenge group received an intranasal inoculation of SARS-CoV-2 Wuhan strain at a dose of 2 × 10^5^ TCID_50_, while the control group received an equivalent volume of physiological saline. Heart and kidney samples were collected at 7 and 9 dpi for histological preparation. All animal experiments were approved by the Institutional Animal Ethics Committee of the Institute of Medical Biology, Chinese Academy of Medical Sciences (Approval No. DWSP202303016) and conducted in accordance with institutional experimental guidelines.

Cell source:The 293T, 16HBE, AC16, and Vero cell lines used in this study were maintained at the Respiratory Virus Laboratory of the Institute of Medical Biology, Chinese Academy of Medical Sciences. All cell lines were regularly authenticated profiling and confirmed mycoplasma-free.

RT–PCR: All primers were synthesized by Qingke Biotechnology (**Supplementary Tables S1**, **S2**). Total RNA was extracted from the samples and transcribed using a reverse transcription kit (Takara, 6215A), after which PCR was performed to determine the sequence characteristics of the sgRNA.

*In vitro* transcription: The target sequence containing the T7 promoter was obtained through PCR amplification (Takara, R045A) and confirmed through sequencing. The sequence was transcribed to mRNA, and a cap and tail (NEB, E2060S) were added, The mRNA was labeled with Cy3(Enzo, ENZ-42505), Cy5 (Enzo, ENZ-42506) and biotin via UTP (Roche, 11388908910).

mRNA transfection: mRNA transfection was performed using Lipofectamine Messenger MAX mRNA Transfection Reagent (Thermo Fisher, LNRNA001). A total of 125 µl of Opti-MEM was used to dilute 7 µl of transfection reagent and 2 µg of mRNA, and the mixture was incubated for 10 minutes and mixed. The mixture then was added to the cells, and the cells were cultured in an incubator.

*In situ* hybridization: The probe used for hybridization was synthesized by BGI (**Supplementary Table S3**). The probe was denatured before the experiment began, and the cell slides were sequentially placed in gradient ethanol solutions for rehydration. Protease K was used to disruptthe cell membrane, gradient ethanol solution was used to dehydrateof the slides, the probe was used to label the ORF3a-E-sgRNA at 4°C, and the nuclei were stained with DAPI. Fluorescence images were captured using aLeica TCS SP8 laser confocalmicroscope.

Flim FRET: Cy3 and Cy5 were incorporated during the mRNA preparation through *in vitro* transcription and used as donors and acceptors, respectively, for *in situ* hybridization and fluorescence staining. The fluorescence lifetimes of Cy3 in the presence of Cy3 only and after the addition of Cy5 were measured and recorded via Leica TCS SP8 laser confocal microscopy in FLIM mode.

Immunohistochemistry: Paraffin-embedded tissue slides were dewaxed with xylene, dehydrated with gradient ethanol, washed with distilled water, subjected to antigen retrieval with sodium citrate, incubated overnight with SARS-CoV-2 N antigen and analyzed using a tissue chemistry section scanner.

SARS-CoV-2 infection: Vero, 16HBE and AC16 cells were cultured in 12-well cell culture plates, and when the cells reached 70-80% confluence, virus maintenance medium containing 2% FBS was used. A total of 1 × 10^6^ TCID50/ml SARS-CoV-2 virus was used for infection, and cell samples were collected at different times during infection. All experiments involving SARS-CoV-2 were conducted in the biosafety cabinet of the biosafety level III facility of the Institute of Medical Biology, Chinese Academy of Sciences.

Western blotting: Cell samples obtained from viral infections and cell transfections were added to RIPA protein lysis buffer supplemented with protease inhibitors and then lysed on ice for 30 minutes. The supernatants were harvested after centrifugation at 12000 rpm and 4°C for 15 minutes. The samples were quantified using a BCA protein assay kit and boiled in 1× SDS buffer at 95°C for 10 minutes. The activation of the host ribosomal pathway was analyzed via protein blotting after viral infection and transfection. The samples were separated by SDS–PAGE, and the proteins were transferred to a 0.22 µm PVDF membrane using a membrane transfer instrument (GenScript). The membrane was incubated with primary antibodies (RPS3, Abcam, Cat# ab128995; eIF4E, Abcam, Cat# ab33768; ORF3a, Abcam, Cat# 280953; 5-mC, Cat# ab214727; IFN-beta, Abcam, Cat# ab275580; and beta-actin, Genetex, Cat# 109639) at 4°C overnight, followed by incubation with secondary antibodies at room temperature for 1 hour. An imaging device was used for band visualization.

Single-cell sequencing: RNA was extracted and reverse transcribed from 16HBE cells infected with SARS-CoV-2 at different time points in the BSL3 laboratory, followed by RNA isolation, library construction, and sequencing on by Singleron Biotechnologies. Among the RNA species produced by infection, the sgRNA is captured specifically by designing probes for the 5’UTR and sgRNA sequences. All RNA seq data were analyzed using R software. Gene Ontology (GO) analysis was performed with the “clusterProfiler” R package 3.16.1. The cell differentiation trajectory was reconstructed using Monocle2. Correlation analysis employed a linear fitting line between ORF3a-E-sgRNA and other sgRNAs or genes to calculate the Pearson correlation coefficient. Cell distribution comparisons between two groups were performed using unpaired two-tailed Wilcoxon rank-sum tests. Comparisons of gene expression or gene signatures between two groups of cells were performed using unpaired two-tailed Student’s t tests. Comparisons of the cell distributions of WH and XBB were performed using paired two-tailed Wilcoxon rank-sum tests. The statistical tests used in the figures are indicated in the figure legends, and statistical significance was set at p < 0.05. The exact values of n are shown in the figures and figure legends.

Probes for single-cell sequencing: three specific probes designed based on the sequence characteristics of the sgRNAs. (1) A 5’ UTR consensus probe capturing all sgRNA types (with subsequent sequencing distinguishing subtypes); (2) an S-sgRNA-specific probe targeting the longest structural gene region where detection efficiency is typically reduced; and (3) an ORF3a-E junction probe specifically detecting nested ORF3a-E-sgRNA transcripts;

### Quantification and statistical analysis

All the statistical analyses were conducted using GraphPad Prism 8 software. The data are presented as the means ± SDs. For comparisons between two groups, a parametric Student’s t test was used. When more than two groups were compared, two-way ANOVA was used. Statistically significant differences are represented in the figures as ∗, ∗∗, ∗∗∗, and ∗∗∗∗ for p values < 0.05, <0.01, <0.001, and <0.0001, respectively.

## Discussion

The emergence of SARS-CoV-2 variants, driven by factors such as immune pressure from vaccination, antiviral drugs, and environmental changes, has led to varying replication and transmission capacities. A thorough understanding of the molecular mechanisms underlying the viral life cycle is important for characterizing viral properties and developing effective vaccines and antiviral strategies. The SARS-CoV-2 genome encodes structural and accessory proteins through the transcription of sgRNAs, which are important links between viral replication and host cell function. However, the discontinuous transcription of these sgRNAs has resulted in sequence diversity, making them challenging to study. There are few relevant studies on SARS-CoV-2 sgRNA at present. Most current studies on SARS-CoV-2 sgRNAs rely on approaches such as the discontinuous transcription of coronaviruses or the use of sequencing techniques to capture 5’ or 3’ ends. These methods may overlook certain sgRNA types because of the limited understanding of their sequence characteristics.

In this study, we identified ORF3a-sgRNA containing a fusion of the ORF3a and E genes during the transcription process. This sgRNA, termed ORF3a-E-sgRNA, was detected at multiple time points during SARS-CoV-2 infection of Vero and 16HBE cells. Most studies capture sgRNAs using probes targeting the conserved 5’UTR, with sgRNA typing achieved through 3’-end sequencing (21, 22). However, ORF3a-sgRNA and ORF3a-E-sgRNA share identical variable fragments (TT) and exhibit substantial sequence overlap. Given that single-cell sequencing typically captures fragments of only approximately 150 bp, these nested transcripts become indistinguishable. According to the literature and our sequence analysis of the 5′ UTRs of existing sgRNAs, no consistent variable RNA fragments in the 5′ UTRs of other sgRNAs have been identified. We also did not detect nested combinations of other sgRNAs encoding structural proteins. This unique composition of the ORF3a-E-sgRNA may represent a more efficient replication mode adopted by SARS-CoV-2 during evolution, allowing large-scale production and self-optimization of sgRNAs to meet the virus’s replication needs and hijack the host translation machinery. We propose two potential pathways for ORF3a-E-sgRNA generation. First, it may arise from the transcription mechanisms of coronaviruses. When the replication-transcription complex (RTC) encounters the transcription regulatory sequence (TRS) upstream of ORF3a, it undergoes discontinuous transcription, producing both ORF3a-sgRNA and ORF3a-E-sgRNA, followed by E-sgRNA. Additionally, E-sgRNA and ORF3a-sgRNA may result from secondary discontinuous transcription events when ORF3a-E-sgRNA is used as a template.

Interestingly, we detected differential expression of sgRNAs, especially ORF3a-E-sgRNA, in cells infected with the Wuhan or XBB SARS-CoV-2 variant. SARS-CoV-2 sgRNAs are generated via discontinuous transcription within the endoplasmic reticulum-Golgi intermediate compartment (ERGIC), with the viral genome used as a template. Consequently, sgRNA expression levels are governed primarily by both the SARS-CoV-2 genome and host-mediated regulation of the ER network. Notably, we observed distinct alterations in the activity of host ribosomes and the endoplasmic reticulum following infection with the Wuhan and XBB variants, which may be a key factors contributing to differences in sgRNA expression, distribution, and the availability of the raw materials required for genome replication More importantly, our findings suggest that ORF3a-E-sgRNA persists throughout the SARS-CoV-2 life cycle, accounting for a relatively high proportion of sgRNAs. ORF3a-E-sgRNA possesses the ability to strongly recruit ribosomes, evade immune responses, and encode viral proteins, all of which are beneficial for efficient viral replication. Moreover, we detected differential expression of ORF3a-E-sgRNA in cells infected with the Wuhan or XBB SARS-CoV-2 variant, suggesting that ORF3a-E-sgRNA may influence infection outcomes in different viral strains. These changes were accompanied by changes in the endoplasmic reticulum, Golgi apparatus, and cellular energy metabolism. IP results demonstrated that both ORF3a-E-sgRNA and ORF3a-sgRNA bind directly and specifically to ribosomal protein S3 (RPS3) at the protein level, with no significant difference in binding affinity between the two. Additionally, both sgRNAs were found to increase RPS3 expression in host cells, indicating a potential indirect regulatory mechanism. Notably, the introduction of an exogenous His-tagged reporter gene did not affect endogenous RPS3 levels. However, expression with either ORF3a-E-sgRNA or ORF3a-sgRNA significantly enhanced RPS3 expression, with ORF3a-E-sgRNA having a more pronounced effect. These findings suggest that RPS3 upregulation may be a secondary result of increased ORF3a translation In summary, in this study, we detected a nested ORF3a-E-sgRNA. Currently, there is limited research on its biological role, but in our opinion, this fusion sgRNA is crucial for virus hijacking of host translation machines and virus replication and release processes during infection, and further exploration is needed.

## Results

### Identification of ORF3a-E-sgRNA during SARS-CoV-2 infection

**Figure 1:**
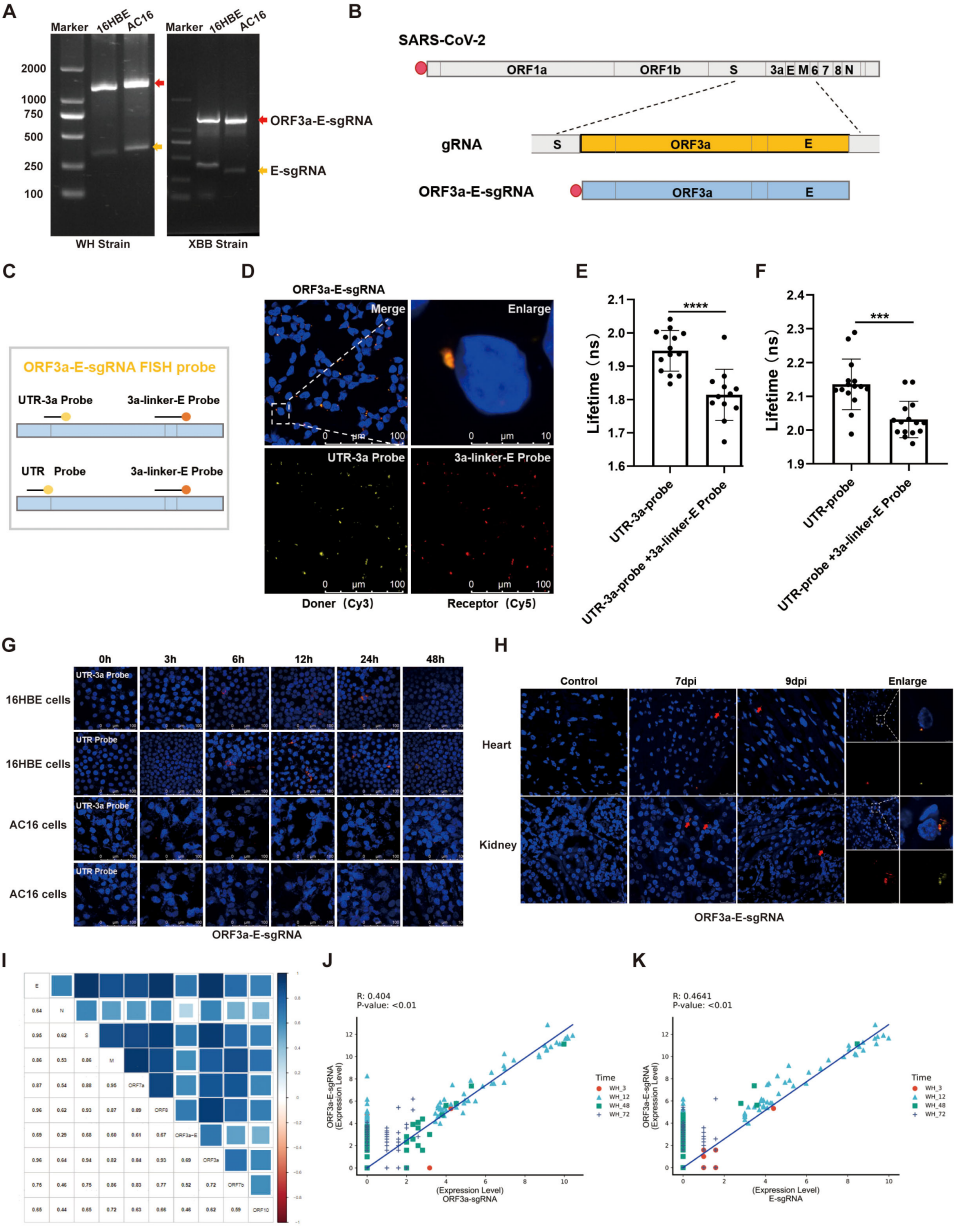
Detection and validation of ORF3a-E-sgRNA during SARS-CoV-2 infection. **(A)** The qualitative detection of ORF3a-E-sgRNA (red arrow) and E-sgRNA (yellow arrow) in viral RNA extracted from16HBE and AC16 cells infected with the Wuhan and XBB strains, at an M.O.I. 0.1, and 24 hpi via RT–PCR. **(B)** Schematic diagram of the sequence characteristics of ORF3a-E-sgRNA. **(C)** Schematic diagram of the probe design for targeting the ORF3a-E-sgRNA sequence. **(D)** Analysis of ORF3a-E-sgRNA-transfected 293T cells using a 3’ fluorescence (Cy3 and Cy5)-labeled probe for *in situ* hybridization, UTR-3a probes, UTR probes and 3a-linker-E probes designed by BGI; a partially enlarged image is shown. Fluorophore colors: Cy3 (yellow), Cy5 (red), DAPI (blue). **(E, F)** Fluorescence resonance energy transfer was used to calculate the fluorescence lifetime of Cy3-UTR-3a probes **(E)** or Cy3-UTR probes as donors **(F)** with or without Cy5-3a-linker-E probes as acceptors for labeling ORF3a-E-sgRNA. *** P < 0.001; **** P < 0.0001. **(G)** Detection of ORF3a-E-sgRNA within 48 hours of SARS-CoV-2 infection in 16HBE and AC16 cells at an M.O.I. of 0.1 via immunofluorescence. **(H)** Detection of ORF3a-E-sgRNA in the heart and kidney tissues of rhesus monkeys on the seventh and ninth days after SARS-CoV-2 infection via immunofluorescence. **(I)** Analysis of the correlations between ORF3a-E-sgRNA and other sgRNAs. **(J, K)** Analysis of the correlation between ORF3a-E-sgRNA and ORF3a-sgRNA **(J)** or E-sgRNA **(K)** from of single-cell sequencing data.

Using primers that bind between the 5’ UTR, the leader core sequence, and the sg-ORF, we investigated various sgRNAs, including M-sgRNA, S-sgRNA, N-sgRNA, and E-sgRNA (**Supplementary Figures S1A, B**). Our analysis revealed a common sequence structure: 5’ UTR (75 bp (ACGAAC) + variable RNA fragment) - ORF - 3’ UTR. Although most of the PCR fragment sizes matched the expected values, we observed an unexpected strong signal for the E-sgRNA that was approximately 800 bp larger than predicted. Sequencing confirmed that this signal represented a fusion sgRNA linking ORF3a with E, resulting in the structure 5’ UTR (75 bp (ACGAAC) + variable RNA fragment) - ORF3a - ORF - medi-E - ORF - 3’ UTR (**Figure 1A**), conforming to the nested nature of CoV sgmRNAs. In theory, the use of common 5’UTR and N-3’ terminal sequences as upstream and downstream primers can detect most nested sgRNAs, but no other nested sgRNAs in addition to the aforementioned fragments were detected in our study. We named this fragment ORF3a-E-sgRNA (**Figure 1B**). Based on the 5’-RACE results for ORF3a-E-sgRNA, we validated its complete structure without extraneous sequences at the 5’ end (**Supplementary Figure S1C**). To detect this molecule accurately, a 3’ fluorescence-labeled probe was designed to target segments of ORF3a-E-sgRNA (**Figure 1C**), and its specificity and sensitivity were confirmed (**Supplementary Figures S1D–F**). The synthesized ORF3a-E-sgmRNA was capped, polyadenylated, and transfected into 293T cells. Fluorescence probing and FLIM-FRET were employed to measure sequence distances, revealing a decrease in the fluorescence lifetime by approximately 0.2 ns upon the addition of the 3a linker E probe (**Figures 1D, E**). This result was also observed when ORF3a-E-sgRNA was detected with another pair of probes (**Figure 1F**). In theory, when donor excitation occurs within <10 nm proximity, energy transfer to the acceptor via FRET reduces the donor’s fluorescence lifetime. This quenching confirms FRET occurrence between Cy3-UTR-3a and Cy5-3a-linker-E labels, demonstrating ORF3a-E-sgRNA presence through molecular proximity. In addition, using this probe, we confirmed the expression of ORF3a-E-sgRNA in different cell lines, such as 16HBE and AC16 cells infected with the Wuhan and XBB strains of SARS-CoV-2 at an M.O.I. of 0.1, as well as in heart and kidney tissues from SARS-CoV-2-infected rhesus monkeys (**Figures 1G, H**). Single-cell sequencing revealed positive correlations between ORF3a-E-sgRNA and other sgRNAs, particularly ORF3a-sgRNA and E-sgRNA (**Figures 1I–K**). Thus, our research confirmed the nested nature of ORF3a-E-sgRNA in the SARS-CoV-2 transcription process and detected the expression of nested sgRNA at different phases in cell lines and tissues infected with different SARS-CoV-2 strains.

### ORF3a-E-sgRNA increases ribosomal subunit protein accessibility and improves the translation efficiency of ORF3a

**Figure 2:**
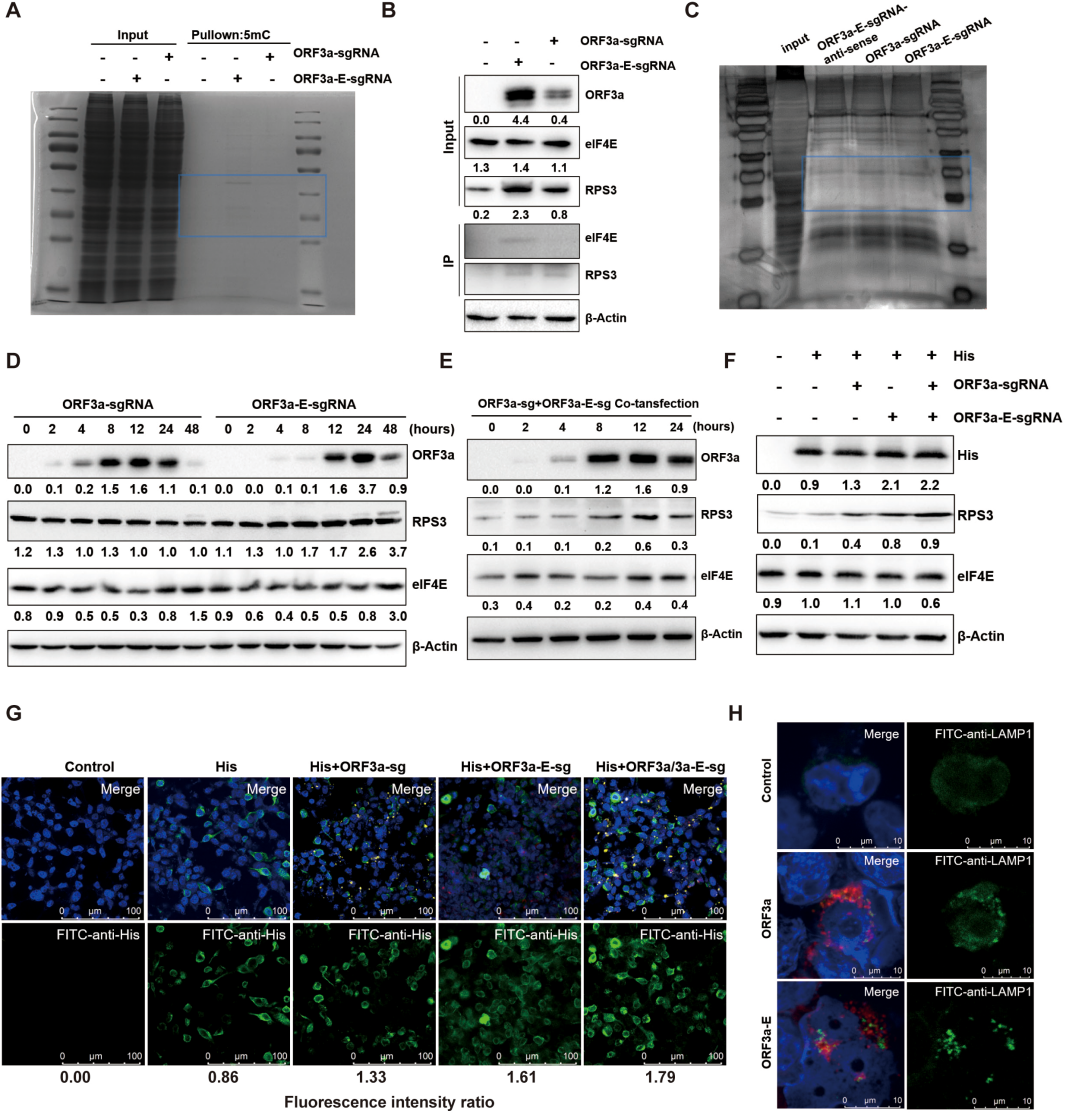
Functional analysis of proteins encoded by ORF3a-E-sgmRNAs. **(A)** After ORF3a-sgmRNA and ORF3a-E-sgmRNA were transfected into 293T cells, the differences in binding proteins were analyzed at the protein level via Coomassie Brilliant Blue staining after IP. Blue area denotes areas of high ribosomal protein expression. **(B)** The analysis of binding of ORF3a-sgmRNA and ORF3a-E-sgmRNA to ribosomal proteins and translation initiation factors in transfected 293T cells using co-immunoprecipitation followed by Western blot. **(C)** Detection of ORF3a-sgmRNAand ORF3a-E-sgmRNA-bound proteins via silver staining from RNA pull-down experiments. Blue area denotes areas of high ribosomal protein expression. **(D)** Comparison of changes in the expression levels of ribosome associated proteins over time in 293T cells transfected with ORF3a-sgmRNA or ORF3a-E-sgmRNAvia Western blotting. **(E)** Changes in the expression of ribosome-associated proteins in 293T cells cotransfected with ORF3a-sgmRNA and ORF3a-E-sgmRNA, Western boltting. **(F)** Detection of the expression of the exogenous reporter gene (His) in 293T cells with or without sgRNA transfection via Western blotting. **(G)** Detection of the fluorescence intensity of His in 293T cells cotransfected with his and sgRNA via immunofluorescence. **(H)** Expression of LAMP1 on the cell membrane surface after the expression of ORF3a-sgmRNA and ORF3a-E-sgmRNA via immunofluorescence.

The SARS-CoV-2 sgmRNA is capped, indicating that its protein translation occurs through a cap-dependent mechanism involving multiple eukaryotic initiation factors, such as the eIF4F complex. We generated sgmRNAs via *in vitro* transcription, capping, and tailing. Immunoprecipitation (IP) and RNA pull-down experiments revealed that nested ORF3a-E-sgmRNA, similar to ORF3a-sgmRNA, can bind to most proteins (**Figures 2A, C**). We continued to detect the translation efficiency of ORF3a-E-sgmRNA, and the IP Western blot results indicated that ORF3a-E-sgmRNA specifically directly binds to the cap-binding eukaryotic translation initiation factor 4E (eIF4E) and the ribosomal protein (RPS3). Interestingly, ORF3a-E-sgmRNA caused greater recruitment of ribosomes, as indicated by the increase in the levels of the ribosomal protein RPS3 (**Figure 2B**). Simultaneously, we observed that ORF3a-E-sgmRNA led to increased expression of the ORF3a protein (**Figure 2B**). Compared with transfection with ORF3a-sgmRNA alone, transfection with ORF3a-E-sgmRNA alone or cotransfection with ORF3a-sgmRNA and ORF3a-E-sgmRNA significantly increased RPS3 levels in host cells, but did not markedly change eIF4E levels (**Figures 2D, E**). These findings suggest that ORF3a-E-sgmRNA has a relatively strong ability to recruit ribosomes. To further investigate the impact of this enhanced ribosome recruitment, we coexpressed an exogenous reporter gene (His) with different sgRNAs. Both Western blot and fluorescence analyses revealed that the translation of the reporter gene (His) was more efficient when it was coexpressed with ORF3a-E-sgmRNA than when it was coexpressed with ORF3a-sgmRNA (**Figures 2F, G**). Moreover, we found that ORF3a proteins expressed by ORF3a-E-sgmRNAs could promote lysosomal exocytosis similar to that of ORF3a-sgmRNAs [as reported in the literature (20)], leading to the increased release of virus particles from infected cells. This was demonstrated by the increased levels of the lysosomal membrane protein LAMP1 on the cell surface (**Figure 2H**). The above results indicate that, compared with ORF3a- sgmRNA, nested ORF3a-E-sgmRNA has a greater ability to impact the host cell translation machinery, supporting increased expression of the ORF3a protein and viral particle release, which are more beneficial for encoding viral proteins during replication.

### Infection with a SARS-CoV-2 variant showed different replication processes related to ribosome activity

**Figure 3:**
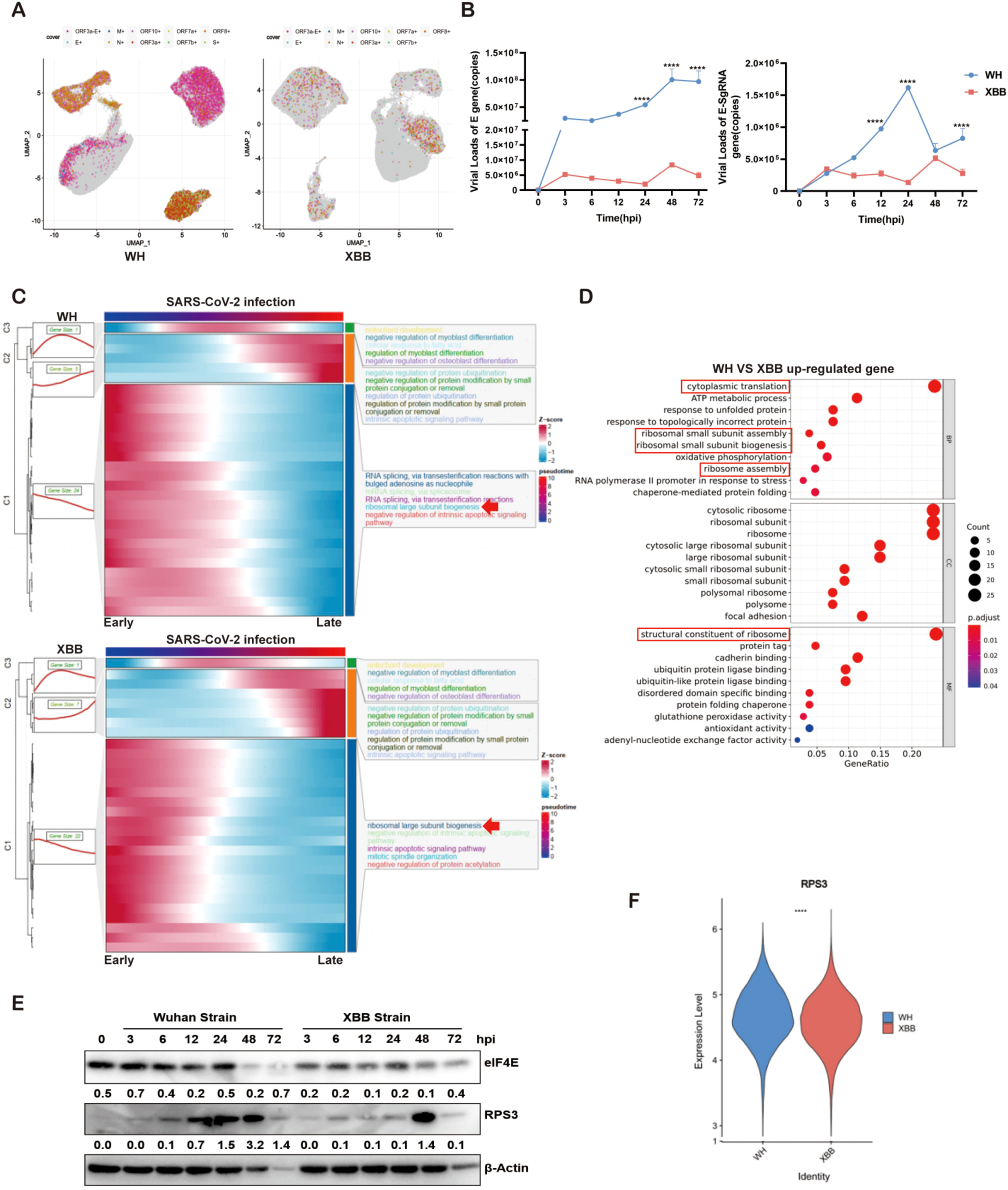
Single-cell sequencing analysis of different variants of SARS-CoV-2. **(A)** Annotation of viral particles in 16HBE cells infected with the Wuhan (left) and XBB (right) strains. **(B)** Detection of E-gRNA and E-sgRNA in 16HBE cells infected with the Wuhan and XBB strains via Q-PCR. **(C)** Differential pathway enrichment in 16HBE cells infected with the Wuhan or XBB strains via pseudotime analysis. **(D)** Comparison of host differentially expressed gene pathway enrichment in 16HBE cells infected with the Wuhan or XBB strain, GO enrichment analysis. **(E)** Comparison of the protein expression levels of the ribosome related factors RPS3 and eIF4E between the Wuhan strain and the XBB strain via Western blotting. **(F)** Comparison of the expression levels of RPS3 in 16HBE cells infected with the Wuhan or XBB strain. **** P < 0.0001.

We examined the functional characteristics of different SARS-CoV-2 strains. Samples from 16HBE cells were collected at various intervals post infection with the 2019 Wuhan prototype strain and the 2022 Omicron variant strain XBB, both at an M.O.I. of 0.1. Single-cell sequencing captured different sgRNA types, including ORF3a-E-sgRNA. Q-PCR and sc-RNA-seq revealed that viral loads increased consistently in both 16HBE cell lines following infection with the Wuhan strain of SARS-CoV-2, whereas the viral load of the XBB strain remained stable over 72 hours. In addition, the viral load generated by the Wuhan strain significantly exceeded that of the XBB strain (**Figures 3A, B**). Moreover, differential gene expression and pathway enrichment analyses revealed that the Wuhan strain maintained a replicative state from the initial to final infection stages, mobilizing ribosomes and the endoplasmic reticulum within the host. In contrast, infection with the XBB strain significantly downregulated ribosome-related pathways (**Supplementary Figures S2**, **S3**). Overall, the host gene expression patterns were similar between infections with the Wuhan and XBB strains in terms of both the infection time and pseudotime series. Notably, ribosomal functional activity was high in the first half of the pseudoperiod (**Figure 3C**). Furthermore, the ribosome-related gene set was highly enriched in hosts infected with the Wuhan strain (**Figure 3D**, **S4**), and further Western Blot experiments also revealed that the translation level of the Wuhan strain was greater than that of the XBB stain (**Figure 3E**). The translation process marked by the 40S ribosomal protein RPS3 was significantly upregulated (**Figure 3F**).

### ORF3a-E-sgRNA is closely related to protein synthesis pathways during SARS-CoV-2 variant replication

**Figure 4:**
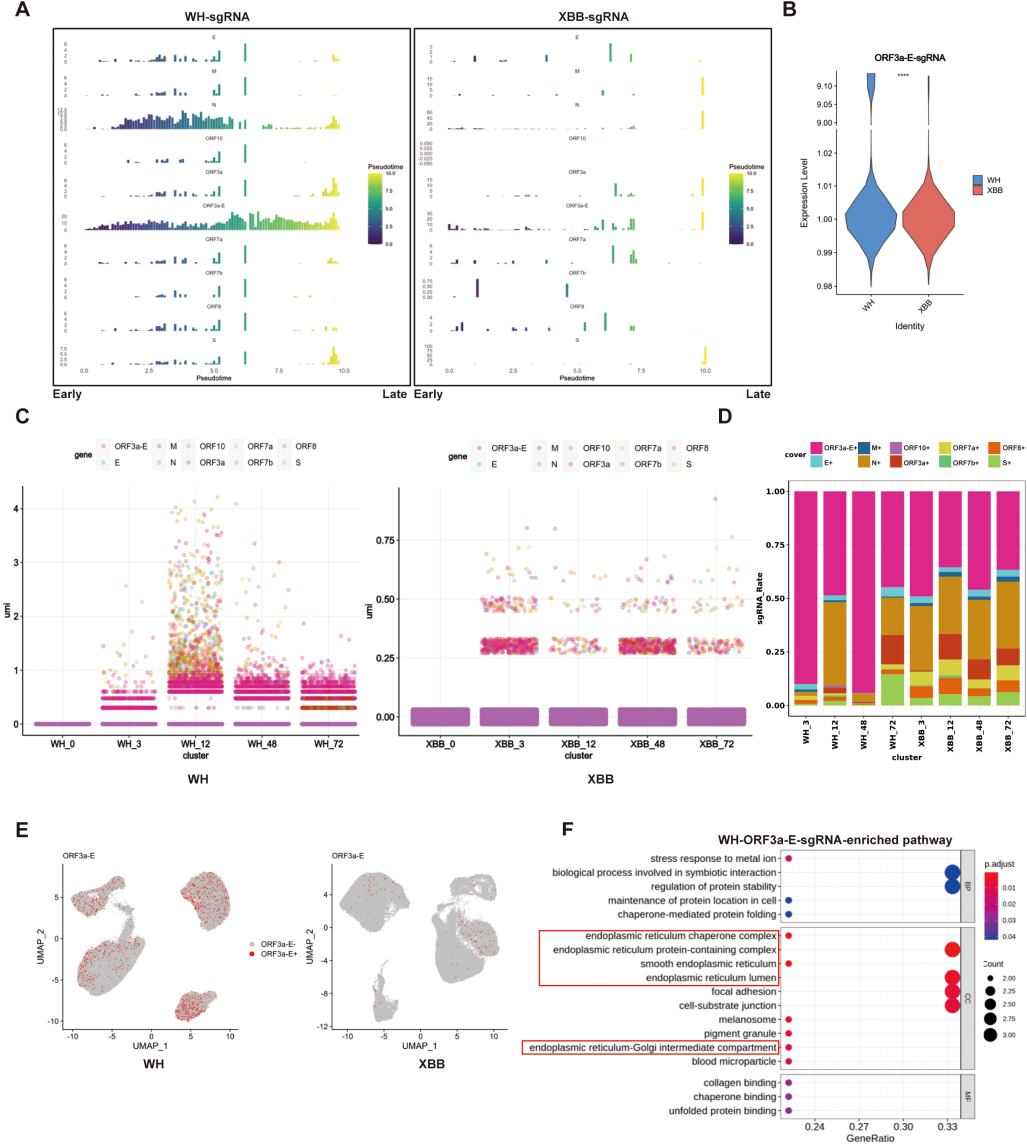
Single-cell sequencing analysis of the functional characteristics of ORF3a-E-sgRNA in different SARS-CoV-2 variants. **(A)** Changes in the proportion of sgRNA in 16HBE cells infected with the Wuhan or XBB strain over pseudotime. The vertical axis represents the abundance of each sgRNA, and the horizontal axis represents infection progression. **(B)** Comparison of the expression ratios of ORF3a-E-sgRNA in Wuhan strain- and XBB strain-infected 16HBE cells. Quantitative analysis of ORF3a-E-sgRNA by single-cell sequencing. **** P < 0.0001. **(C)** Changes in the proportion of sgRNA in 16HBE cells infected with the Wuhan or XBB strain over the infection period. The vertical axis shows the detection rates from single-cell sequencing, and the horizontal axis represents hours post-infection. **(D)** Comparison of the expression ratios of sgRNA in Wuhan strain- and XBB strain-infected 16HBE cells. **(E)** Annotation of the ORF3a-E-sgRNA in 16HBE cells infected with the SARS-CoV-2 Wuhan strain or the XBB strain separately. **(F)** GO enrichment analysis of ORF3a-E-sgmRNAs in 16HBE cells infected with the Wuhan strain.

We further analyzed the dynamics of SARS-CoV-2 sgRNAs during infection via both pseudotime and infection timing approaches. The pseudotime analysis simulated the SARS-CoV-2 infection cycle based on changes in gene expression during different stages of infection, dividing it into early, middle, and late stages. We annotated the dynamic changes in different sgRNA types as the pseudoinfection time varied (**Supplementary Figure S5**). The results revealed that the expression proportion of Wuhan sgRNAs dynamically changed as pseudotime progressed, with their distribution being relatively concentrated in the first half of the pseudoperiod. Among them, ORF3a-E-sgRNA was present throughout the entire pseudoinfection period. In contrast, the distribution of XBB sgRNA was scattered and lacked an obvious pattern (**Figures 4A, B**). The infection timing results further revealed that ORF3a-E-sgRNA was the most abundant among all the sgRNAs during infection with the Wuhan strain. However, this ORF3a-E-sgRNA activity was relatively weak after XBB strain infection (**Figures 4C, D**).

We further investigated the functional effects of ORF3a-E-sgRNA ondifferent SARS-CoV-2 variants. Compared with Wuhan strain infection, targeted analysis of sgRNAs using sc-RNA and functional enrichment revealed that most sgRNAs were not enriched in host pathways during XBB strain infection (**Supplementary Figures S6–S8**). However, the ORF3a-E-sgRNA from the Wuhan strain was enriched in pathways related to protein synthesis, such as protein folding, ER-Golgi transport, and protein stability (**Figures 4E, F**). Overall, the activity of ORF3a-E-sgRNA appears to be a key factor underlying the differences in host protein synthesis between SARS-CoV-2 variants.

### ORF3a-E-sgRNA limits the innate immune response for effective replication of SARS-CoV-2

**Figure 5:**
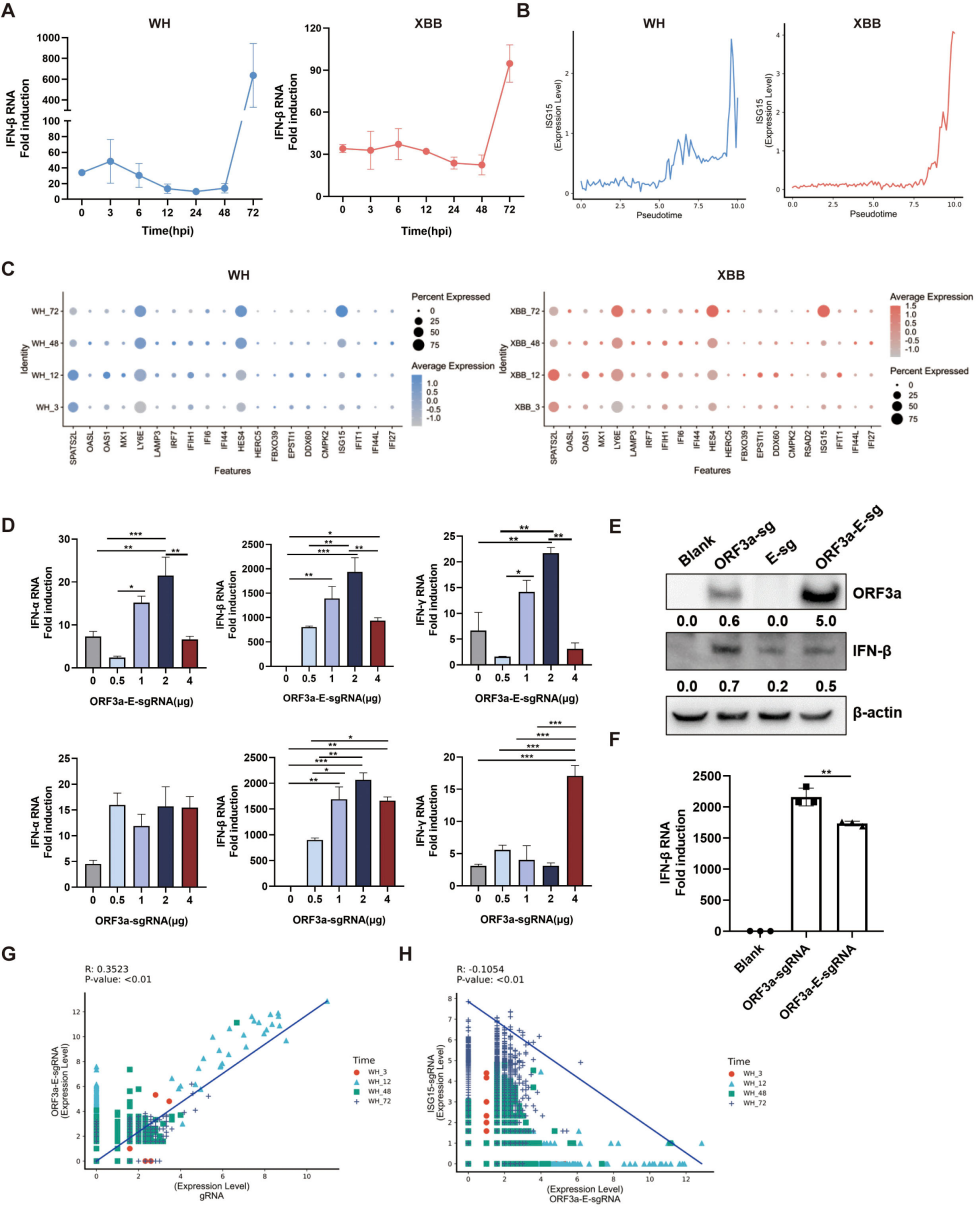
Ability of ORF3a-E-sgRNA to respond during the SARS-CoV-2 life cycle. **(A)** IFN-β expression in 16HBE cells infected with the Wuhan or XBB strain over the infection period, as determined via Q-PCR. **(B)** ISG15 expression in 16HBE cells infected with the Wuhan or XBB strain over pseudotime. **(C)** Differential expression levels of the interferon-stimulated genes at 3, 12, 48 and 72 hpi in 16HBE cells infected with the Wuhan or XBB strain over the infection period. **(D)** Measurement of the expression levels of IFN-α,IFN-β and IFN-γ in 293T cells transfected with different amounts of ORF3a-sgRNA or ORF3a-E-sgRNA via Q-PCR. *P < 0.05; **P < 0.01; ***P<0.001. **(E)** Comparison of the protein expression levels of IFN-β in 293T cells transfected with ORF3a-sgRNA or ORF3a-E-sgRNA via Western blotting. **(F)** Comparison of IFN-β transcription levels in 293T cells transfected with ORF3a-sgRNA or ORF3a-E-sgRNA. ** P < 0.01. **(G, H)** Analysis of the correlations between ORF3a E-sgRNA and genomic RNA **(G)** and between ORF3a E-sgRNA and ISG15 **(H)** single-cell sequencing data.

The structural and nonstructural proteins of SARS-CoV-2 play crucial roles in viral evasion of the host antiviral immune response, primarily by targeting key molecules in the IFN-I signaling pathway. This process may also involve ORF3a-E-sgRNA and its high expression during SARS-CoV-2 infection. We observed that in 16HBE cells infected with the Wuhan or XBB strain, the expression levels of interferon-beta (IFN-β) and the interferon-stimulated gene ISG15 remained low within the first 48 hours but then rapidly increased at 72 hours, potentially leading to an inflammatory cytokine storm (**Figures 5A, B**). Further analysis revealed that the expression levels of many interferon-stimulated genes increased significantly as the infection progressed, especially at 72 hours (**Figure 5C**). This inverse correlation between sgRNA expression and the immune response suggests the presence of innate immune antagonists, such as ORF6 and ORF9b. To verify the immune evasion ability of ORF3a-E-sgRNA, we transfected it into 293T cells. The expression levels of IFN-α, IFN-β, and IFN-γ increased at certain transfection doses but decreased at higher doses (**Figure 5D**). Importantly, compared with transfection with ORF3a-sgRNA alone, transfection with ORF3a-E-sgRNA resulted in greater stronger immune evasion ability (**Figures 5E, F**). Moreover, the ORF3a-E-sgRNA level was positively correlated with the genomic RNA level but negatively correlated with the expression of the interferon-stimulated gene ISG15 (**Figures 5G, H**). In summary, high-level expression of ORF3a-E-sgRNA maintains low host innate immunity during the SARS-CoV-2 life cycle, enabling effective viral replication and release.

## Data Availability

Raw and processed data are available on CNGB Nucleotide Sequence Archive (CNSA: https://db.cngb.org/cnsa) with accession number CNP0007728.
