# Supplementary material for: A fusion ORF3a-E subgenomic RNA involved in SARS-CoV-2 infection efficacy by influencing cellular protein synthesis
Source: Front Immunol. 2025 Aug 15;16:1619538. doi: 10.3389/fimmu.2025.1619538 (PMC12395053; doi:10.3389/fimmu.2025.1619538)
Supplement: Supplementary file 1 [file DataSheet1.docx]

**ORF3a-E fusion subgenomic RNA involves in SARS-CoV-2 infecting efficacy by influencing cellular protein synthesis**

**Contents**

[Figure Supplement 1 Verification of Sequence Characteristics of sgRNA and detection of ORF3a-E-sgRNA. 2](#_Toc184197341)

[Figure Supplement 2 Analysis of upregulation pathway enrichment in 16HBE cells infected with SARS-CoV-2 Different Variants. 3](#_Toc184197342)

[Figure Supplement 3 Analysis of downregulation pathway enrichment in 16HBE cells infected with SARS-CoV-2 Different Variants. 5](#_Toc184197343)

[Figure Supplement 4 Comparison of upregulation pathway in cells infected with different SARS-CoV-2 strains. 6](#_Toc184197344)

[Figure Supplement 5 Pseudo temporal analysis of the SARS-CoV-2 infection cycle. 7](#_Toc184197345)

[Figure Supplement 6 Annotation of the different sgRNA types in 16HBE cells infected with different SARS-CoV-2 strains. 8](#_Toc184197346)

[Figure Supplement 7 Functional analysis of SARS-CoV-2-Wuhan sgRNA. 9](#_Toc184197347)

[Figure Supplement 8 Functional analysis of SARS-CoV-2-XBB sgRNA. 10](#_Toc184197348)


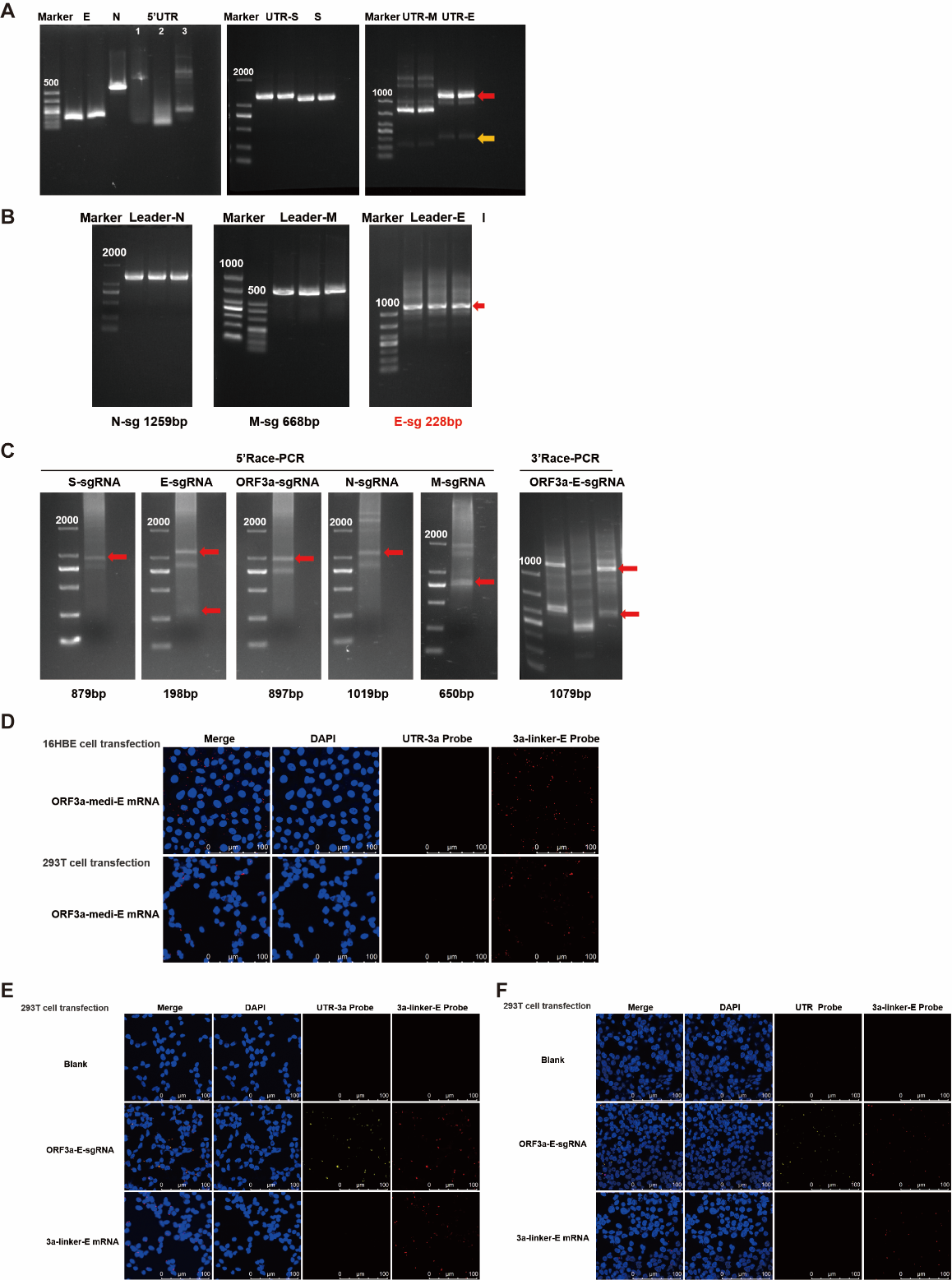


Figure Supplement 1 Verification of Sequence Characteristics of sgRNA and detection of ORF3a-E-sgRNA.

(A-B) Validation of the discontinuous transcription of sgRNAs from vero cells infected with SARS-CoV-2 at MOI 0.1 through the RT-PCR primers between 5'UTR or the leader core sequence and sg-ORF; (A) ORF3a-E-sgRNA (red arrow) and E-sgRNA (yellow arrow); (B) The red arrow indicates the target sgRNA. (C) Detection of the presence of other sequences before and after the 5 'and 3' ends of the ORF3a-E-sgRNA through Race PCR. (D) Specificity and sensitivity detection of overlapping regions between ORF3a-E-sgRNA and genomic RNA labeled with UTR-3a probe. (E-F) ORF3a-E-sgRNA transfected to 293T cells labeled by UTR-3a probe or UTR probe in situ.


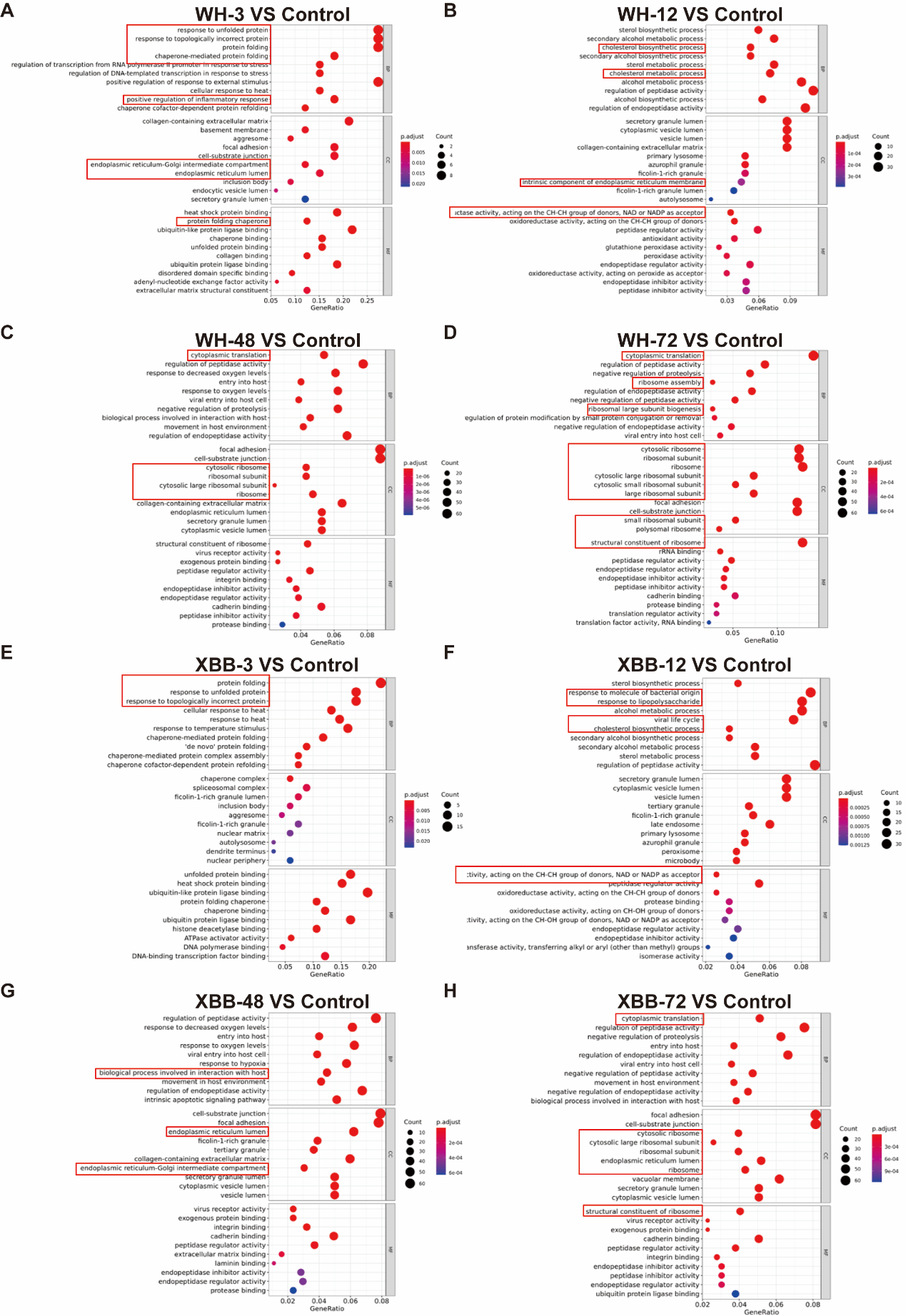


Figure Supplement 2 Analysis of upregulation pathway enrichment in 16HBE cells infected with SARS-CoV-2 Different Variants.

(A-D) The up-enriched GO terms were revealed in SARS-CoV-2-Wuhan infected 16HBE cells at 3 hpi, 12 hpi, 48 hpi, 72 hpi compared with control. (E-H) The up-enriched GO terms were revealed in SARS-CoV-2-XBB infected 16HBE cells at 3 hpi, 12 hpi, 48 hpi, 72 hpi compared with control. All of the above have been calculated for P-values and have statistical significance.


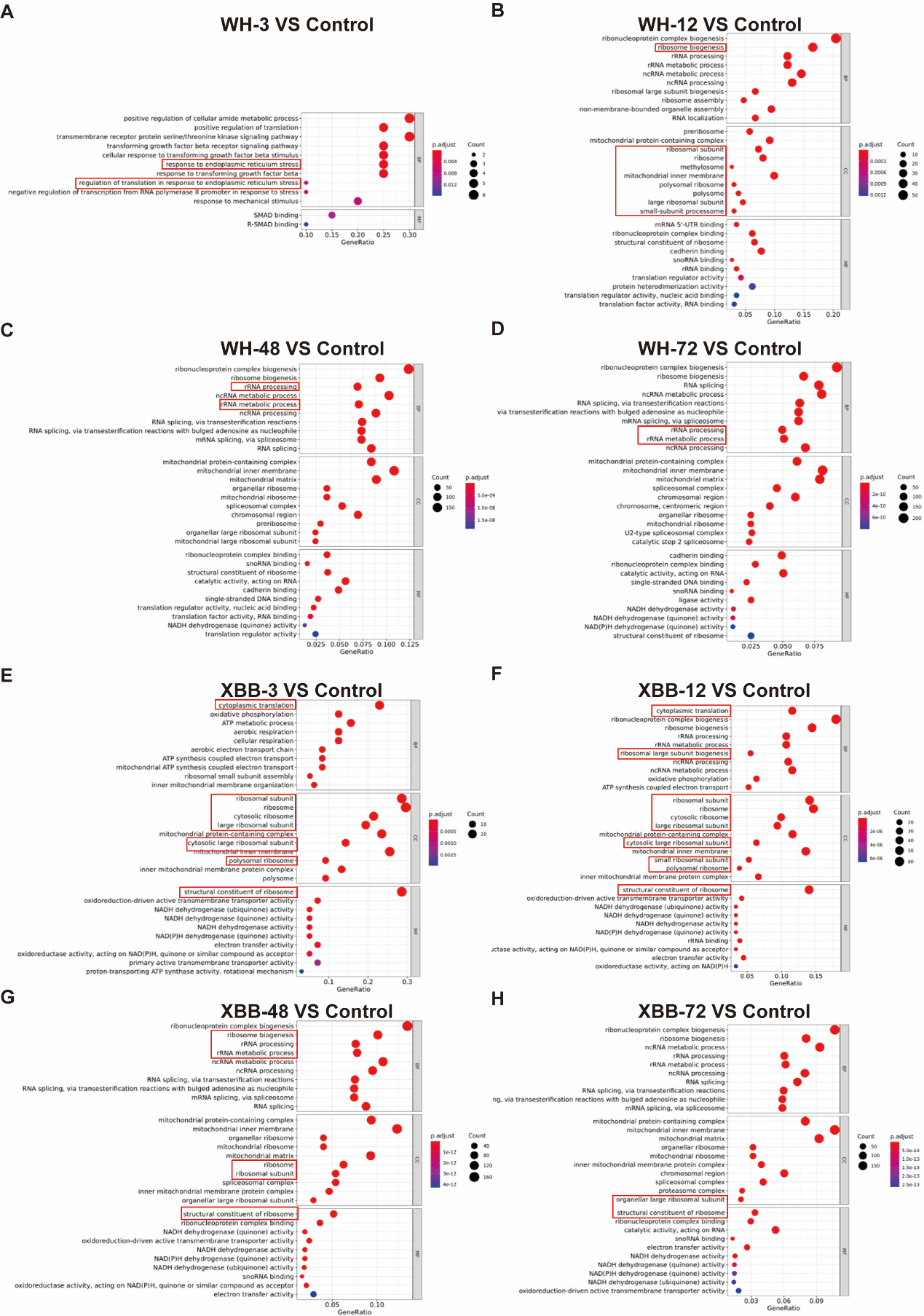


Figure Supplement 3 Analysis of downregulation pathway enrichment in 16HBE cells infected with SARS-CoV-2 Different Variants.

(A-D) The down-enriched GO terms were revealed in SARS-CoV-2-Wuhan infected 16HBE cells at 3 hpi, 12 hpi, 48 hpi, 72 hpi compared with control. (E-H) The down-enriched GO terms were revealed in SARS-CoV-2-XBB infected 16HBE cells at 3 hpi, 12 hpi, 48 hpi, 72 hpi compared with control. All of the above have been calculated for P-values and have statistical significance.


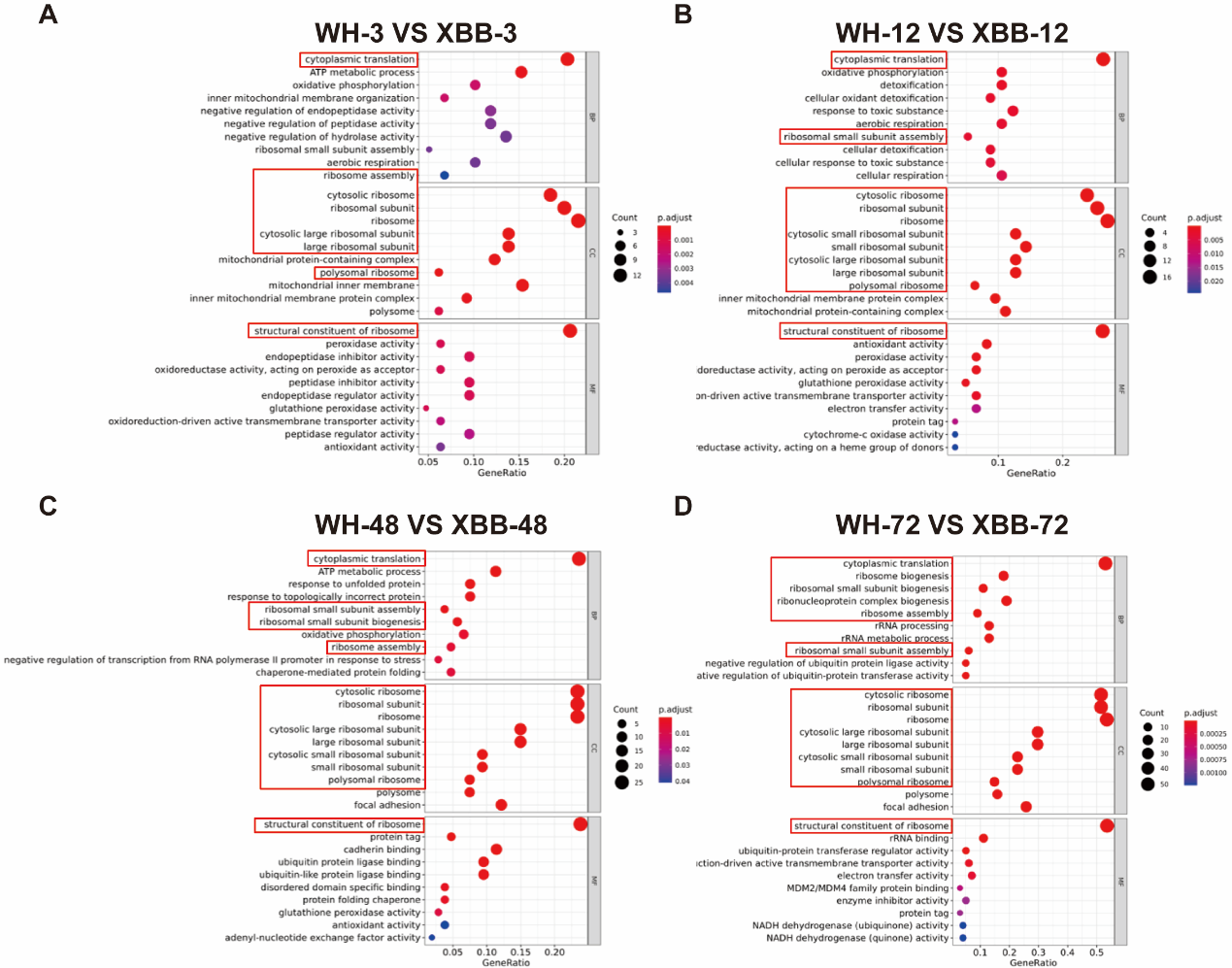


Figure Supplement 4 Comparison of upregulation pathway in cells infected with different SARS-CoV-2 strains.

Comparison of upregulation pathway enrichment analysis of 16HBE cells infected with SARS-CoV-2 Wuhan and XBB strain after 3hpi (A), 12hpi (B), 48hpi (C) and 72hpi (D), and calculating the p-value. P<0.05 is statistically significant.


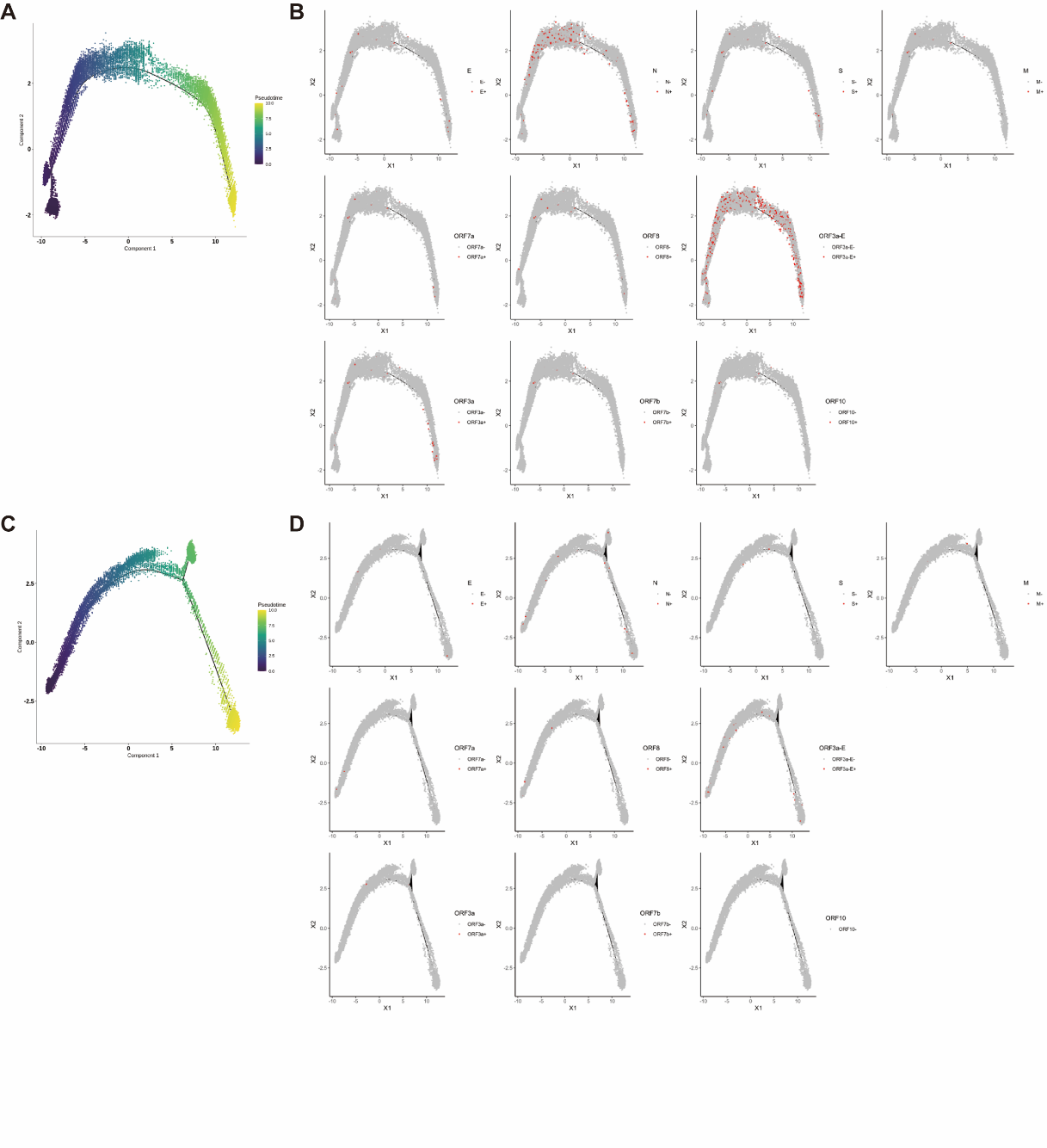


Figure Supplement 5 Pseudo temporal analysis of the SARS-CoV-2 infection cycle. (A and C) Constructing the SARS-CoV-2 infection cycle using monocle based on the changes in gene expression within the 16HBE host cells infected with SARS-CoV-2 Wuhan strain (A) and XBB strain (C). (B and D) Annotated pseudo temporal directional trajectory maps of SARS-CoV-2 Wuhan strain (B) and XBB strain (D) with different sgRNA types.


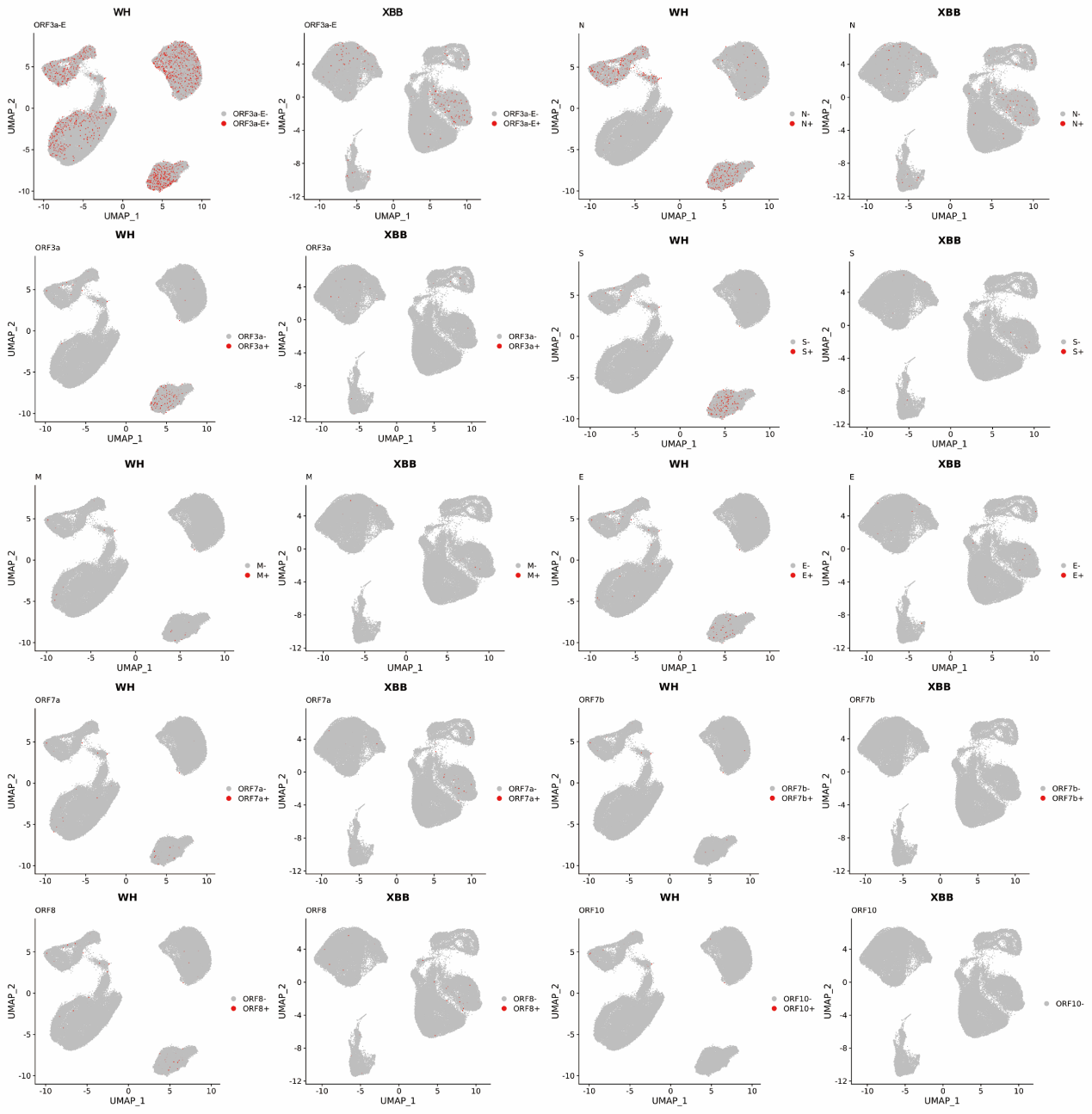


Figure Supplement 6 Annotation of the different sgRNA types in 16HBE cells infected with different SARS-CoV-2 strains.

Annotation of the different sgRNA types in 16HBE cells infected with SARS-CoV-2 Wuhan strain and XBB strain separately, and dividing the cells into single cells with and without sgRNA.


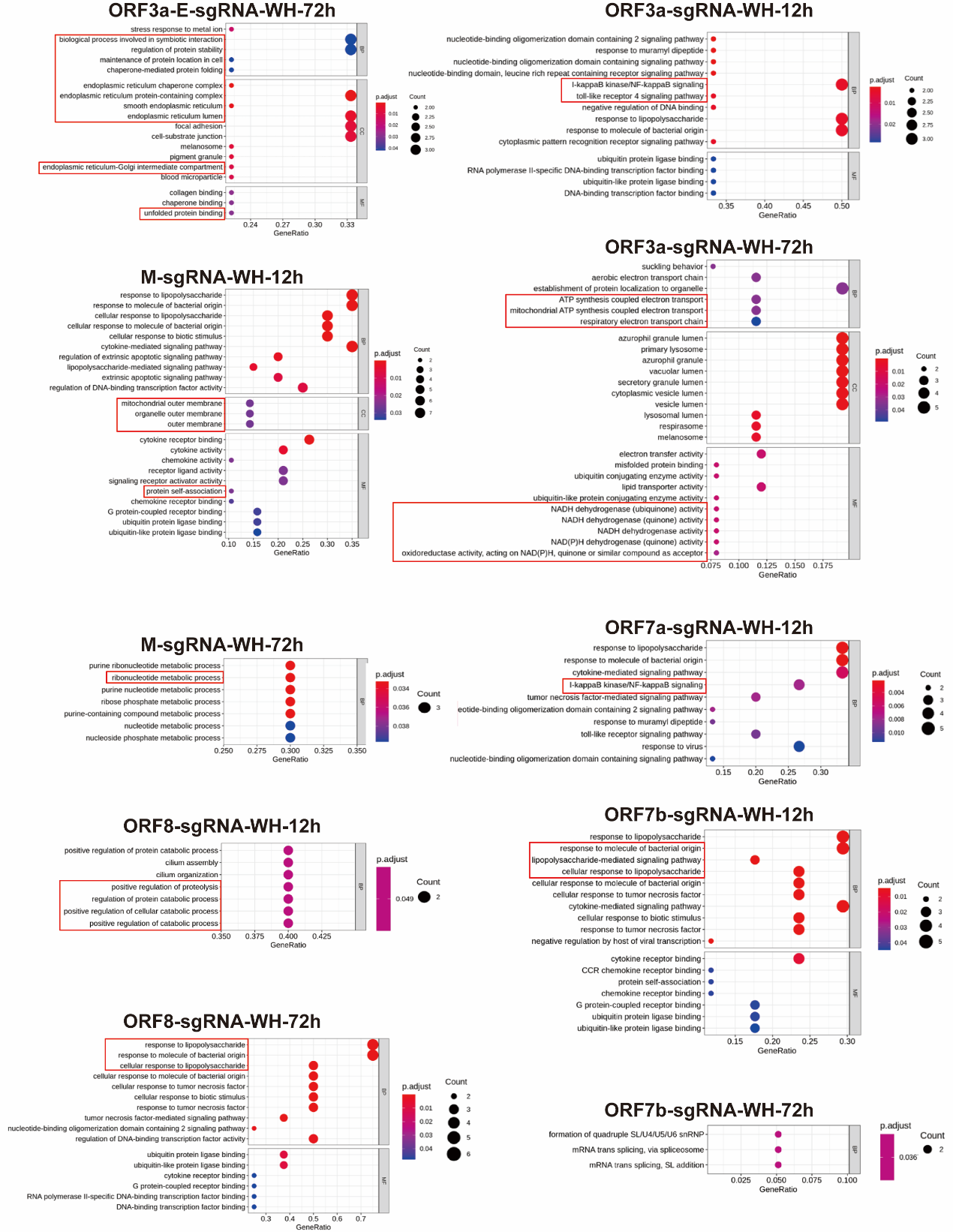


Figure Supplement 7 Functional analysis of SARS-CoV-2-Wuhan sgRNA. Functional enrichment analysis of cells containing and not containing sgRNAs in SARS-CoV-2-Wuhan strain infection, and calculating the p-value. P<0.05 is statistically significant.


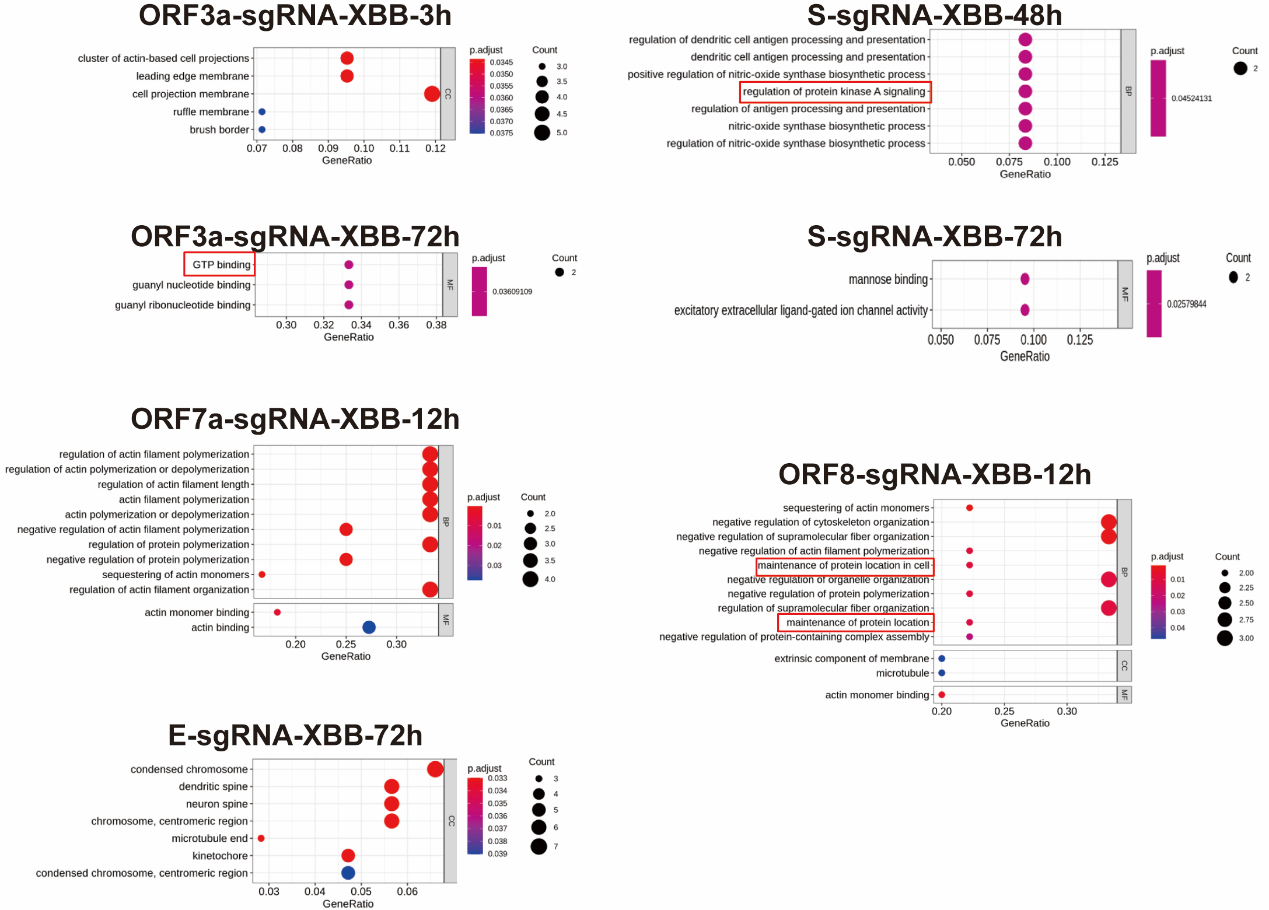


Figure Supplement 8 Functional analysis of SARS-CoV-2-XBB sgRNA.

Functional enrichment analysis of cells containing and not containing sgRNAs in SARS-CoV-2-XBB strain infection, and calculating the p-value. P<0.05 is statistically significant.
